# Regulation of the Transferrin Receptor Recycling in Hepatitis C Virus-Replicating Cells

**DOI:** 10.3389/fcell.2020.00044

**Published:** 2020-02-11

**Authors:** Vanessa Haberger, Fabian Elgner, Jessica Roos, Daniela Bender, Eberhard Hildt

**Affiliations:** ^1^Division of Virology, Paul Ehrlich Institute, Langen, Germany; ^2^Division of Safety of Medical Products and Devices, Paul Ehrlich Institute, Langen, Germany; ^3^German Center for Infection Research (DZIF), Braunschweig, Germany

**Keywords:** α-taxilin, hepatitis C virus, transferrin receptor, iron metabolism, HCV superinfection

## Abstract

After binding of its ligand transferrin, the transferrin receptor (TfR) is internalized via early endosomes. Ligand and receptor can be recycled. α-Taxilin was identified as an essential factor for TfR recycling. Apart from its role for iron uptake, TfR is a coreceptor for hepatitis C virus (HCV) infection. In HCV-replicating cells, the amount of a-taxilin is decreased. This study aims to investigate the effect of decreased α-taxilin levels in HCV-replicating cells on recycling of TfR, its amount on the cell surface, on iron uptake, and the impact of a disturbed TfR recycling on HCV superinfection exclusion. TfR amount and localization were determined by CLSM and surface biotinylation. α-taxilin expression was modulated by CRISPR-Cas9 knockout, siRNA, and stable or transient overexpression. For analysis of HCV superinfection fluorophor-tagged reporter viruses were used. The amount of α-taxilin is decreased in HCV-infected cells. In accordance to this, the protein amount of TfR is significant lower in HCV-positve cells as compared to the control, while TfR expression is not affected. Due to the impaired recycling, internalized TfR is degraded by the endosomal/lysosomal system. The significant lower number of TfR molecules on the cell surface is reflected by reduced transferrin binding/internalization and strong reduction of intracellular iron level. Overexpression of α-taxilin in HCV-replicating cells rescues TfR recycling, augments TfR on the cell surface, and restores transferrin binding. The block of superinfection in HCV-replicating cells could be overcome by overexpression of α-taxilin. Taken together, the diminished level of α-taxilin in HCV-replicating cells prevents recycling of TfR leading to decreased transferrin binding and iron uptake. Disappearance of TfR from the cell surface could be a factor contributing to the exclusion of superinfection by HCV.

## Introduction

The hepatitis C virus (HCV) causes acute and chronic liver diseases. Currently, about 71 million people worldwide suffer from chronic infection with HCV and approximately 399,000 people die each year on HCV-associated liver cirrhosis and hepatocellular carcinoma (WHO, 2019). Although the recent development of a new generation of HCV-specific effective drugs, the direct acting antivirals (DAAs), mark a significant progress to treat and to cure HCV the number of HCV-infected patients is still increasing. This underlines the urgent need for the development of an effective vaccine against HCV. Moreover, there is still a variety of open questions with respect to the HCV life cycle.

Hepatitis C virus belongs to the genus Hepacivirus of the family *Flaviviridae*. HCV as a flavivirus has a single-stranded, positive-sensed RNA genome that encompasses 9.6 kb and consists of a 5′-untranslated region (UTR), a long open reading frame encoding for a single polyprotein of about 3010 aa and a 3′-UTR (Choo et al., 1989). The highly structured 5′ and 3′ UTRs harbor conserved structures required for viral replication. The internal ribosomal entry site (IRES) of the 5′-UTR binds the 40S ribosomal subunit and initiates the translation of the viral genome in a cap-independent manner (Pisarev et al., 2005). After translation, the polyprotein is cleaved by cellular and viral proteases into 10 mature proteins: the structural proteins core that forms the viral capsid and the envelope proteins E1 and E2; the viroporin p7 and the non-structural (NS) proteins, NS2, NS3, NS4A, NS4B, NS5A, and NS5B (Appel et al., 2006). The NS proteins form the replicase complexes that are sufficient for the genome replication. HCV-genome replication can take place in the absence of HCV structural proteins. This allows the generation of subgenomic replicons that allow the analysis of HCV genome replication in the absence of infectious virion production (Bartenschlager et al., 2013).

Through interaction with several receptors of hepatocytes, the virus enters the cell. Scavenger receptor B1 (SRB1), syndecan-1, syndecan-4, and heparan sulfate proteoglycan (HSPG) are required for attachment of viral particles and infection (Dao Thi et al., 2012; Shi et al., 2013; Lefèvre et al., 2014). Additionally, the tetraspanin CD81, claudin-1 (CLDN1) and occludin (OCLN), low density lipoprotein receptor (LDLR), as well as the cholesterol transporter Niemann-Pick C1-like protein (NPC1L1) are also required for the HCV entry (Pileri et al., 1998; Evans et al., 2007; Ploss et al., 2009; Albecka et al., 2012; Sainz et al., 2012). Another coreceptor mediating the virus entry is the transferrin receptor (TfR) with a monomer size of 90–95 kDa (Daniels et al., 2006; Martin and Uprichard, 2013). The ubiquitously expressed TfR1 is required for iron uptake into cells. Iron ions bind to the extracellular ligand transferrin. Subsequently, the endocytic adaptor protein, TfR1 trafficking protein (TTP), facilitates the internalization of the TfR-transferrin complex via clathrin-mediated endocytosis (Tosoni et al., 2005). By lowering pH value in early endosomes, iron ions dissociate from the ligand and are finally released into the cytoplasm (Dautry-Varsat et al., 1983). TfR can be recycled to maintain a fast cycle of iron uptake for the cell. After receptor recycling, the iron-free apotransferrin remains bound to TfR and is released into the extracellular space with neutral pH (Dautry-Varsat et al., 1983). Beside receptor recycling, TfR can be degraded in lysosomes (Maxfield and McGraw, 2004).

α-Taxilin consists of 546 amino acids and belongs to the taxilin family (Nogami et al., 2004). Taxilin family members bind to members of the syntaxin family, which are involved in intracellular vesicle transport by formation of SNARE (soluble *N*-ethylmaleimide-sensitive fusion protein-attachment protein receptor) complexes. Thus, α-taxilin interacts with syntaxin-1a, syntaxin-3, as well as syntaxin-4. Exclusively, free syntaxins that are not part of the SNARE complex can bind to α-taxilin (Nogami et al., 2003). Thus, α-taxilin exerts an inhibitory effect on the SNARE-complex formation and thereby on SNARE-dependent transport processes. Knockdown of α-taxilin was found to be associated with lysosomal degradation of TfR and impaired recycling of TfR. Further analyses revealed that sorting nexin 4 (SNX4) is involved in the recycling of TfR and interacts with α-taxilin (Sakane et al., 2014).

In HCV-replicating cells, the amount of α-taxilin is strongly reduced while the level of syntaxin-4 is increased. Syntaxin-4-dependent exocytosis is involved in the release of HCV (Elgner et al., 2016a). Through a reduced amount of α-taxilin by HCV, a higher amount of free syntaxin-4 is available to assemble the tSNARE complex. In accordance to this, the release of HCV particles is impaired by overexpression of α-taxilin and enhanced by silencing the α-taxilin expression (Elgner et al., 2016a). Vice versa, overexpression of syntaxin-4 favors HCV release, whereas silencing inhibits particle release (Ren et al., 2017).

Here, we investigate the effect of the decreased level of α-taxilin in HCV-replicating cells on the recycling of TfR, its amount on the cell surface, the capacity to bind transferrin and on the iron uptake, and the impact on HCV superinfection.

## Materials and Methods

### Cell Culture

The highly permissive Huh7-derived cell line Huh7.5 (Blight et al., 2002), Huh7.5 with a stable α-taxilin overexpression (Huh7.5-Taxilin), and Huh7.5 α-taxilin knockdown (127706-1-2) (Hoffmann et al., 2013) were used for the electroporation of HCV RNA. The Huh7-derived cell line Huh7.5.1 was used for generating CRISPR-Cas9 α-taxilin knockout clones.

All cell lines were grown in Dulbecco’s modified Eagle’s medium (DMEM; Sigma) supplemented with 10% fetal calf serum, 2 mM L-glutamine, 100 units/mL penicillin and 100 μg/mL streptomycin (complete DMEM) at 37°C, 5% CO_2_, and 90% humidity. For Huh7.5-Taxilin, harboring the pDest26-TXLNA, 750 μg/mL G418 (Geneticin, Millipore) was added to complete DMEM for selection of clones. For selection of Huh7.5 α-taxilin knockdown cells complete DMEM with 5 μg/mL Puromycin (Sigma) was used.

The HBV-permissive cell line HepaRG was used for generating CRISPR-Cas9 α-taxilin knockout clones. Cells were grown in Williams’ Medium E (Sigma) supplemented with 10% fetal calf serum, 100 units/mL penicillin and 100 μg/mL streptomycin, 5 μg/mL insulin, and 50 μM hydrocortisone.

### Viability Assay

Cells were seeded in a 96-well plate with a density of 1 × 10^4^ cells/200 μL. Viability was measured using the lactate dehydrogenase (LDH) Cytotoxicity Detection Kit (Takara, Clontech), according to the manufacture’s protocol. Triton X-100 was used as a positive control for cytotoxicity. Absorbance was measured with a Microplate Reader (Tecan).

### Plasmids

Plasmids pFK-JFH1/GND (Wakita et al., 2005) as replication-deficient variant, pFK-Jc1 (Pietschmann et al., 2006), and pFK-Jc1-E1mC (Jc1 E1R) (Elgner et al., 2016b) have been described previously. pDest26-TXLNA was described by Elgner et al. (2016a). pFK-Jc1-NS5AGFP (Jc1 5AG) was generated by amplification of the EGFP sequence using plasmid pEGFP N1 (clontech) as a template. The amplified insert with overhangs complementary to the NS5A sequence and the plasmid pFK-JHF1/J6 were digested with the enzymes *Rsr*II und *Hin*dIII. After dephosphorylation of the plasmid with Antarctic Phosphatase (NEB), the amplified fragment was ligated into the plasmid with T4 DNA Ligase (Thermo Scientific). Plasmid Rab9-RFP was kindly provided by Prof. Höning (Universität Köln, Germany).

### *In vitro* Transcription and Electroporation

*In vitro* transcription (IVT) of plasmid DNA and electroporation of HCV RNA were performed as described previously (Lohmann et al., 2001). For IVT, the T7 Scribe^TM^ Standard RNA IVT Kit (Biozym) was used according to the manufacturer’s protocol.

### Bafilomycin and Bortezomib Treatment

At 72 h after electroporation, cells were treated with 50 nM Bafilomycin A1 (BFLA, Sigma) or 10 nM Bortezomib (Selleckchem) for 16 h for inhibition of late stage autophagy.

### Double Infection of Huh7.5 and Huh7.5-Taxilin Cells With HCV

Huh7.5 and Huh7.5-Taxilin cells were transfected with Jc1 E1R- or with Jc1 5AG-RNA by electroporation. The construct Jc1 E1R is coding for a fusion protein of E1 and mCherry. The second construct Jc1 5AG is coding for a fusion protein of NS5A and eGFP. After 48 h of electroporation, Jc1 E1R Huh7.5 cells were incubated with infectious supernatant of Jc1 5AG cells for additional 48 h, followed by fixation with FA (4%). Nuclei were stained with DAPI and analysis was performed at the CLSM (confocal laser-scanning microscope) for detection of the mCherry- and eGFP-specific fluorescence.

### Real-Time PCR

RNA isolation of cell lysates and cDNA synthesis were performed as described by Ploen et al. (2013). Real-Time PCR was performed as described by Masoudi et al. (2014) with the following primers: JFH1-fwd (5′-ATG ACC ACA AGG CCT TTC G-3′), JFH1-rev (5′-CGG GAG AGC CAT AGT GG-3′), TfR fwd (5′-TGA AGA GAA AGT TGT CGG AGA AA-3′), TfR rev (5′-CAG CCT CAC GAG GGA CAT A-3′), txlna fwd (5′-ATG AAG AAC CAA GAC AAA AAG A-3′), txlna rev (5′-CTG GCT GCT GCC GGG AC-3′), hRPL27cDNA fwd (5′-AAA GCT GTC ATC GTG AAG AAC-3′), and hRPL27cDNA rev (5′-GCT GCT ACT TTG CGG GGG TAG-3′). RPL27 (ribosomal protein L27) was used for normalization.

### Transient Transfection and Silencing of Gene Expression

Hepatitis C virus-negative Huh7.5 cells were transfected 4 h after seeding either with 50 nM α-taxilin specific siRNA or scrRNA (sc-39644 and sc-37007, Santa Cruz Biotechnology) using N-TER^TM^ Nanoparticle siRNA Transfection System (Sigma) according to the manufacturer’s protocol. For controls, cells were again transfected after 24 h of transient RNAi transfection with empty plasmid pUC18 or pDest26-TXLNA using PEI (polyethyleneimine; Polyscience). Cells were harvested three days post-transfection with siRNA.

### CRISPR-Cas9 Knockout of α-Taxilin in HepaRG and Huh7.5.1 Cells

The Plasmid pSpCas9(BB)-2A-Puro (PX459) V2.0 was a gift from Feng Zhang (Addgene plasmid # 62988^1^; RRID:Addgene_62988). DNA oligos txlna_sg_fwd: 5′-CACCGAACCAAGACAAAAA GAACG-3′ and txlna_sg_rev: 5′-AAACCGTTCTTTTTGTC TTGGTTC-3′ or off-target_sg_fwd: 5′-CACCGCACTACCA GAGCTAACTCA-3′ and off-target_sg_rev: 5′-AAACTGAGTT AGCTCTGGTAGTGC-3′ were annealed, phosphorylated, and ligated into the vector PX459 according to the instructions from the Zhang lab (Le Cong et al., 2013; Ran et al., 2013). The resulting plasmid px459-txlna was transfected into HepaRG and Huh7.5.1 cells using polyethylenimine. Transfected cells were selected for 2 weeks, starting 48 h post-transfection with 0.5 μg/mL Puromycin supplemented in the HepaRG growth medium (Williams’ E medium including 10% fetal calf serum, 100 units/mL penicillin, 100 μg/mL streptomycin, 50 μM hydrocortisone, and 5 μg/mL insulin) or DMEM complete supplemented with 5 μg/mL Puromycin, respectively. Knockdown of α-taxilin was confirmed by Western blot with lysates of selected monoclonal colonies.

### Antibodies

The following commercial antibodies were used: anti-transferrin-receptor (H68.4, Thermo Scientific), anti-α-taxilin (H-66, Santa Cruz Biotechnology), anti-α-taxilin (atlas antibodies, Sigma), anti-HCV-NS3 (8G-2, Abcam), anti-LAMP-2/Cd107b (AF6228, R&D Systems), anti-EGFR (EP38Y, abcam), anti-LDLR (Thermo Scientific), and anti-β-actin (AC-74, Sigma). For the recycling kinetic anti-TfR (TFRC/1818, Biozol) was used. For detection of HCV NS5A, a polyclonal rabbit-derived serum was used (Bürckstümmer et al., 2006). IRDye^®^ 800CW and IRDye^®^ 680RD secondary antibodies for Western blotting were obtained from LI-COR. For immunofluorescence staining, Alexa Fluor 488-conjugated secondary antibodies (Invitrogen) as well as Cy3- and Cy5-conjugated secondary antibodies (Jackson Immuno Research Laboratories, Inc.) were used.

### Fluorescent Dyes

Transferrin-488 and transferrin-555 were obtained from Invitrogen and DAPI (4,6-diamidino-2-phenylindol) was used from Carl Roth.

### SDS-PAGE and Western Blot Analysis

Huh7.5 cells were lysed in RIPA buffer [50 mM Tris-HCl pH 7.2, 150 mM NaCl, 0.1% SDS (w/v), 1% sodium deoxycholate (w/v), 1% Triton X-100]. Cell lysates were sonicated, debris separated by centrifugation, and protein amount measured by Bradford assay. After subsequent SDS-PAGE and Western blotting, immunostaining was quantified using the Odyssey Imaging System (LI-COR). If indicated in the figure legend, total protein staining kit (LI-COR) was used as loading control.

### Biotinylation Assay

Biotinylation assays with cleavable sulfo-NHS-SS-biotin to obtain biotinylated cell surface proteins were performed as described by Sakane et al. (2014). Biotinylated proteins were precipitated with Pierce^TM^NeutrAvidin^TM^ agarose beads (Thermo Scientific) according to the manufacturer’s protocol “Batch Method for Immunoprecipitation.” Binding Buffer consisting of phosphate-buffered saline (PBS) and 1% NP-40, as recommended, was used. Biotinylated TfR was eluted by boiling the beads 20 min in 1x SDS-PAGE sample buffer. To prevent stable dimers of TfR, concentration of ß-mercaptoethanol was increased to 20% (v/v).

### Indirect Immunofluorescence Analysis

Analysis of indirect immunofluorescence was performed as described by Ploen et al. (2013). Immunofluorescence staining was analyzed using a CLSM (confocal laser-scanning microscope, 510 Meta, Carl Zeiss) and ZEN 2009 software. The objective used was 100x (NA, 1.46). Quantification of fluorescence intensity was quantified with ZEN 2009 software. The fluorescence intensity of AF488-labeled transferrin (transferrin-488), LAMP2, and TfR was determined using the Software Zen 2009 (Zeiss).

### Transferrin Recycling Assay

Analysis of the recycling of the TfR-transferrin-488 or TfR-transferrin-555 complex was performed as described by Sakane et al. (2014). The incubation was performed at 37^*o*^C. CLSM was used for analysis after fixation of the cells. Cells were treated with BFLA (50 nM) for 1 h.

### Recycling Kinetic

To compare HCV + and HCV-Huh7.5 cells, cells were electroporated with Jc1 and GND and stained 72 hpe. Cells were washed with PBS and detached with Accutase at 37°C. The cells were transferred into reaction tubes for centrifugation at 400 *g*, 10 min, 4°C. The cell pellet was resuspended in complete DMEM with 20 μg/mL of holo-transferrin/mL. The incubation with holo-transferrin was performed for 20 min at 37°C. Again, cells were centrifuged at 400 g, 5 min, 4°C. Non-specifically bound holo-transferrin was stripped from the cell surface (1 min, pH 3.0 PBS). Then, cells were incubated in DMEM complete for different time points up to 25 min at 37°C. At each time point, cells were washed and stored on ice to stop the internalization process. For labeling, cells were incubated with 2 μg/mL TfR primary antibody in cold serum-free DMEM for 30 min on ice followed by two wash steps at 4^*o*^C with PBS. For detection of the bound antibody cells were incubated 30 min with Alexa Fluor 488-conjugated secondary antibody at 4°C. After staining, cells were washed with cold PBS, fixed in 2% FA, and re-suspended in PBS for flow cytometry measurement (BD FACS LSRII SORP).

### Iron Quantification

Iron was determined by the ferrozine method using the IRON2 test that was run on a Cobas 8000 c701system (Roche).

### Neutralization-Assay to Block HCV Superinfection

Huh7.5 cells were seeded in a 12-well plate and incubated 20 min at 37°C with 5 μg/mL primary antibody binding to the extracellular domain of TfR. The HBV-specific MA/18/7 served as control. After binding at the cell surface unbound antibodies were washed away with PBS. A mixture of either HCV infectious supernatant and TfR- antibodies or of HCV infectious supernatant and MA/18/7-antibodies were added to the Huh7.5 cells and incubated for 36 h. Cells were fixed with 4% FA and stained with NS5A-specific antibody for CLSM analysis.

### Statistical Analysis

Results are described as means ± standard errors of the means (SEMs) from at least three experiments. The significance of the results was analyzed by two-tailed Student’s *t*-test, using GraphPad Prism 7 software for Windows. The bars in the figures represent SEMs. In histograms showing relative changes compared with the control, the control group was arbitrarily set as 1. Here, a SEM for the control group cannot be reported, as standardization of the measured values (relative to the control group) was performed for each of the independent assays. Therefore, measurements for the treatment groups in each assay were dependent (matched). Statistical significance is represented in figures as follows: ^∗^*P* < 0.05, ^∗∗^*P* < 0.01, ^∗∗∗^*P* < 0.001, ^****^P < 0.0001.

## Results

### The Amount of TfR Is Reduced in α-Taxilin Deficient Cells

**FIGURE 1:**
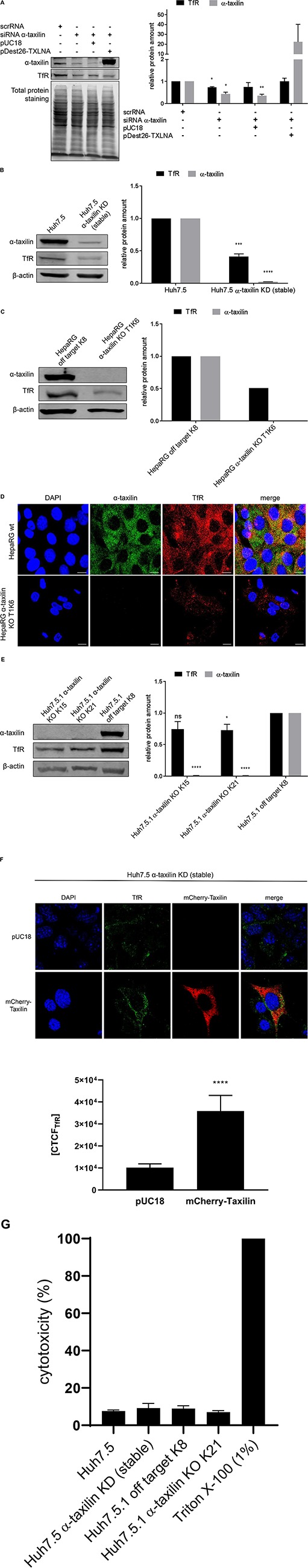
Reduced amounts of the TfR in α-taxilin knockdown (KD) and α-taxilin knockout (KO) cells. **(A)** (Left) Western blot analysis of cellular lysates derived from Huh7.5 cells transfected with an α-taxilin-specific siRNA (50 nM) or scrRNA (50 nM) (control). 24 h after siRNA transfection, the cells were transfected with pDest26-TXLNA (1 μg) or pUC18 (1 μg) (control) to rescue the α-taxilin KD. The cells were analyzed 72 h after siRNA transfection using α-taxilin- and TfR-specific antibodies. Total protein staining served as a loading control (lower panel). (Right) Densitometric quantification of α-taxilin and the TfR from two independent experiments. scrRNA transfected cells served as a control and were set as 1 (*n* = 2; mean ± SEM). Two-tailed unpaired *t*-test, *p < 0.05, **p < 0.01. **(B)** (Left) Western blot analysis of cellular lysates derived from Huh7.5 cells and stable α-taxilin KD Huh7.5 cells (Huh7.5 α-taxilin KD) using α-taxilin- and TfR-specific antibodies. ß-actin served as a loading control. (Right) Densitometric quantification of α-taxilin and the TfR from three independent experiments. Huh7.5 cells served as a control and were set as 1 (*n* = 3; mean ± SEM). Two-tailed unpaired *t*-test, ***p < 0.001, ****p < 0.0001. **(C)** (Left) Western blot analysis of cellular lysates derived from HepaRG cells (HepaRG off target K8) and stable α-taxilin KO HepaRG cells (HepaRG α-taxilin KO T1K6) using α-taxilin- and TfR-specific antibodies. ß-actin served as a loading control. (Right) Densitometric quantification of α-taxilin and the TfR from one experiment. HepaRG cells (HepaRG off target K8) served as a control and were set as 1 (*n* = 1). **(D)** CLSM analysis of HepaRG wt and stable α-taxilin KO HepaRG cells (HepaRG α-taxilin KO T1K6) using α-taxilin (green) and TfR (red)-specific antibodies. Nuclei were stained with DAPI (blue). Laser intensity and digital gain were kept constant for HepaRG wt and HepaRG α-taxilin KO (HepaRG α-taxilin KO T1K6) cells. Magnifications, x100. Scale bar represents 10 μm. **(E)** (Left) Western blot analysis of cellular lysates derived from Huh7.5.1 cells (Huh7.5.1 α-taxilin off target K8) and stable α-taxilin KO Huh7.5.1 cells (Huh7.5.1 α-taxilin KO K15/K21) using α-taxilin- and TfR-specific antibodies. ß-actin served as a loading control. (Right) Densitometric quantification of α-taxilin and the TfR from three independent experiments. Huh7.5.1 cells (Huh7.5.1 off target K8) served as a control and were set as 1 (*n* = 3; mean ± SEM). Two-tailed unpaired *t*-test, *p < 0.05, ****p < 0.0001, ns = not significant. **(F)** (Top) The amount of TfR in Huh7.5 α-taxilin KD (stable) cells can be rescued by transient transfection with an α-taxilin mCherry plasmid (red). Huh7.5 α-taxilin KD (stable) cells were stained using a TfR-specific antibody (green). Nuclei were stained with DAPI (blue). Laser intensity and digital gain were kept constant. Magnifications, x100. (Bottom) Quantification of the fluorescence intensity of TfR (green) in Huh7.5 α-taxilin KD (stable) cells transfected with the α-taxilin mCherry plasmid and the pUC18 control compared to the transfected Huh7.5 cells. Total fluorescence per cell was calculated using ImageJ software and the following formula: corrected total cell fluorescence (CTCF) = integrated density - (area of selected cell × mean fluorescence of background readings). In total, a minimum of 10 cells were measured (mean ± SEM). Two-tailed unpaired *t*-test, ****p < 0.0001. **(G)** Viability assay for Huh7.5 and α-taxilin deficient cells. LDH release was measured by a microplate reader to determine cytotoxicity. Treatment with Triton X-100 (1%) served as a positive control.

The α-taxilin-dependent recycling of TfR in Huh7.5 cells was analyzed based on a transient α-taxilin knockdown using siRNA. The knockdown of α-taxilin in Huh7.5 cells results in a significantly decreased amount of α-taxilin and of TfR as compared to the cells transfected with scrRNA (Figure 1A). After rescue of the α-taxilin expression by transfection of the siRNA α-taxilin treated cells with an expression vector encoding for α-taxilin (pDest26-TXLNA), the amount of TfR was restored. Transfection with pUC18 of the siRNA α-taxilin treated cells served as control.

These data were confirmed using a stable α-taxilin knockdown in Huh7.5 cells (Figure 1B) and a knock out by CRISPR-Cas9 in HepaRG cells (Figures 1C,D) as well as in Huh7.5.1 cells (Figure 1E). Again, a significant reduction in the amount of TfR was found in the α-taxilin-deficient cells as compared to the control cells (Figures 1B–E).

To control the specificity of the observed effects, rescue experiments were performed. For this purpose, the Huh7.5 cells harboring a stable α-taxilin knock down were transfected with an expression vector encoding the mcherry-Taxilin fusion protein and the amount of TfR was analyzed by confocal immunofluorescence microscopy using a TfR-specific antibody. For a more detailed analysis, the amount of TfR was quantified by determination of the CTCF value (Figure 1F). In order to exclude unspecific effects due to knock down/out, viability assays were performed with Huh7.5 off target K8 cells, Huh7.5.1 α-taxilin KO21cells, Huh7.5 α-taxilin KD stable, and Huh7.5 cells. The viability assays show that knock down or knock out of α-taxilin does not affect the cell viability of Huh7.5 cells (Figure 1G). These data indicate that silencing of α-taxilin expression in Huh7.5 and HepaRG cells leads to a specifically decreased amount of the TfR.

### The Amount, but Not the Expression of TfR Is Decreased in HCV-Replicating Cells

Apart from its role in iron uptake, the TfR is a coreceptor for HCV (Martin and Uprichard, 2013) and therefore involved in HCV entry. As in HCV-replicating cells the amount of α-taxilin is decreased, it was analyzed whether the recycling and the amount of TfR is affected.

**FIGURE 2:**
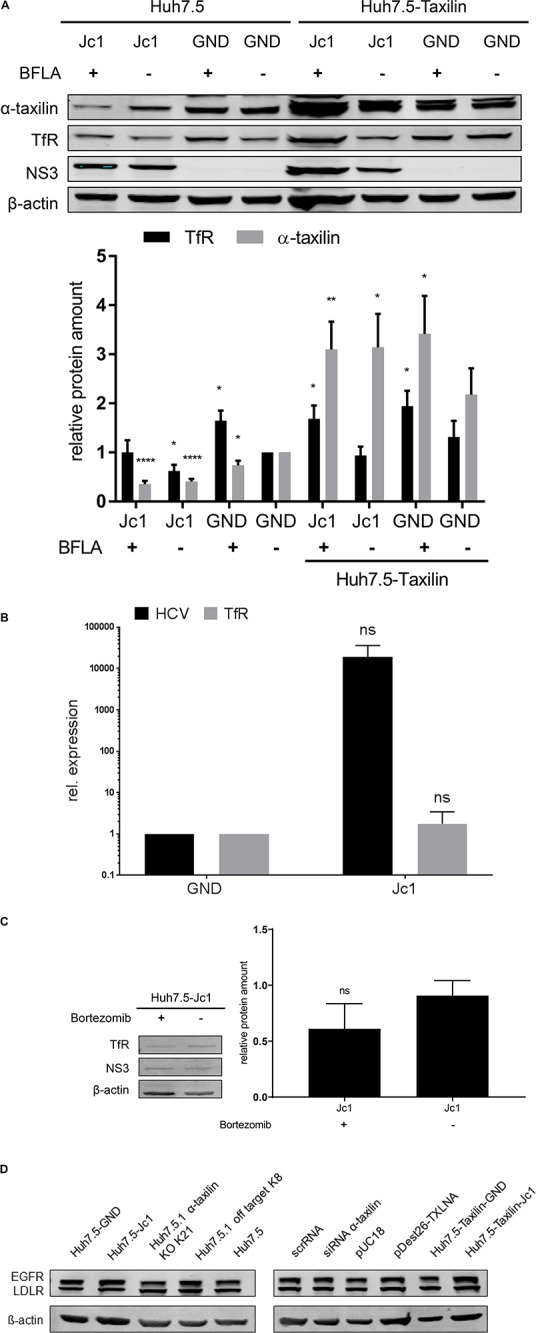
The reduced amount of the TfR in HCV-positive cells is not due to a decreased expression. **(A)** (Top) Western blot analysis of cellular lysates derived from HCV-positive (Jc1) and HCV-negative cells (GND) Huh7.5 and stably α-taxilin overexpressing Huh7.5 cells (Huh7.5-Taxilin)72 hpe, treated with BFLA (50 nM) for 16 h. For detection, α- taxilin-, TfR-, and NS3-specific antibodies were used. ß-actin served as a loading control. (Bottom) Densitometric quantification of α-taxilin and the TfR from seven independent experiments. Untreated HCV-negative cells (GND) served as a control and were set as 1 (*n* = 7; mean ± SEM). Two-tailed unpaired *t*-test, *p < 0.05, **p < 0.01, ****p < 0.0001. **(B)** RT-qPCR analysis of TfR transcripts in HCV-positive (Jc1) and HCV-negative (GND) cells. HCV-negative cells served as a control and were set as 1 (*n* = 3, mean ± SEM). The samples were normalized to RPL27 transcripts. Two-tailed unpaired *t*-test, ns = not significant. **(C)** Western blot analysis of cellular lysates derived from HCV-positive (Jc1) Huh7.5 cells 72 hpe, treated with Bortezomib (10 nM) for 16 h. For detectionTfR-, NS3-specific antibodies were used. ß-actin served as a loading control. Densitometric quantification of α-taxilin and the TfR from three technical replicates. Untreated HCV-positive cells (Jc1) served as a control and were set as 1 (*n* = 3; mean ± SEM). Two-tailed unpaired *t*-test, ns = not significant. **(D)** Western blot analysis of cellular lysates derived from HCV-positive (Jc1) or negative (GND) Huh7.5 cells or Huh7.5 α-taxilin KD (stable) cells 72 hpe. For detection EGFR-, and LDLR-specific antibodies were used. ß-actin served as a loading control. Densitometric quantification of α-taxilin and the TfR from three technical replicates. Untreated HCV-positive cells (Jc1) served as a control and were set as 1 (*n* = 3; mean ± SEM).

Western blot analysis of cellular lysates derived from HCV-positive cells (Jc1) revealed a significant decrease in the amount of α-taxilin and of TfR as compared to the HCV-negative control (GND) (Figure 2A).

To investigate whether the decreased amount of TfR in HCV-replicating cells is due to a reduced expression, the TfR-specific mRNA was quantified by qPCR. The qPCR revealed that the amount of TfR-specific transcripts is not significantly changed in HCV-replicating cells (Jc1) as compared to the HCV-negative control (GND, Figure 2B).

To study whether the decreased level of the TfR in HCV-positive cells can be rescued by overexpression of α-taxilin, Huh7.5 cells stably overexpressing α-taxilin (Huh7.5-Taxilin) were electroporated with HCV RNA (Jc1). The Western blot analysis shows that overexpression of α-taxilin in HCV-replicating cells indeed restores the amount of the TfR (Figure 2A).

Taken together, these data indicate that the decreased amount of α-taxilin in HCV-replicating cells is causative for the lower amount of the TfR in these cells.

As there is no effect on the expression of the TfR, it was investigated whether the reduced amount of the TfR in HCV-replicating cells indeed is due to an impaired recycling and subsequent autophagosomal/lysosomal degradation. In order to study this, the lysosomal activity was blocked by BFLA and the effect on the amount of TfR was analyzed by Western blot. The Western blot shows that the HCV-dependent decrease of the TfR amount is abolished, if the cells were treated with BFLA. Compared to untreated cells the level of the TfR increases, while the amount of α-taxilin is not affected (Figure 2A).

In contrast to this, western blot analysis of cellular lysates derived from HCV-positive cells treated with the proteasomal inhibitor bortezomib revealed no increase in the amount of TfR (Figure 2C).

To investigate whether other receptors that are subjected to recycling processes and relevant for HCV entry the effect of HCV replication and of α-taxilin knock down on the amount of EGFR or LDLR was investigated. The western blot shows that neither HCV infection nor α-taxilin knock down has an effect on the amount of EGFR or LDLR (Figure 2D).

These data indicate that the decreased amount of TfR in HCV-replicating cells is not due to an impaired expression. The decrease reflects an impaired recycling that directs the TfR to the lysosomal compartment, where it is degraded.

### In HCV-Replicating Cells BFLA Leads to an Accumulation of TfR

**FIGURE 3:**
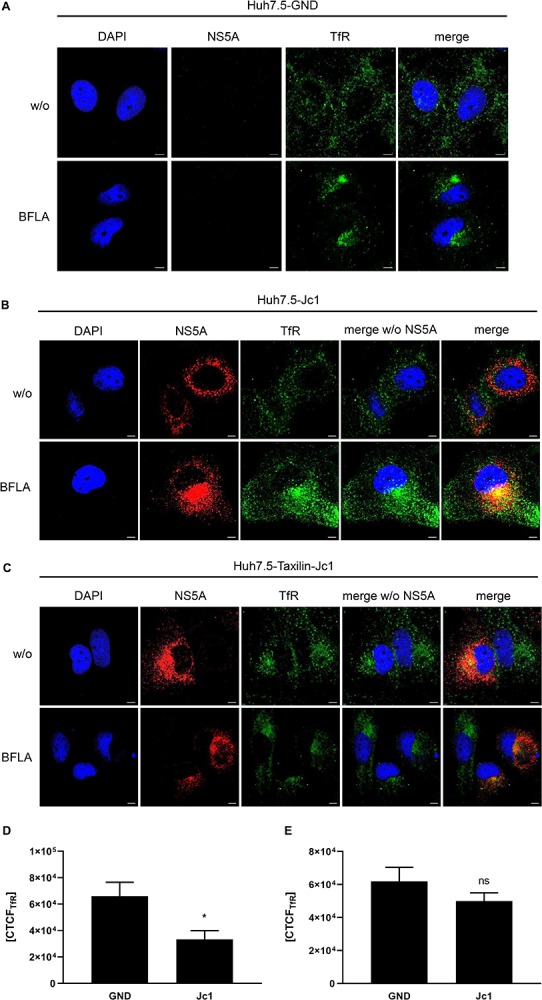
Bafilomycin A leads to an accumulation of TfR in HCV-replicating cells. **(A)** CLSM analysis of HCV-negative Huh7.5 cells (GND) treated with BFLA (50 nM) for 16 h. The cells were stained with TfR (green) and NS5A-specific (red) antibodies. Nuclei were stained with DAPI (blue). Laser intensity and digital gain were kept constant for untreated and BFLA-treated cells. Magnifications, x100. Scale bar represents 5 μm. **(B)** CLSM analysis of HCV-positive Huh7.5 cells (Jc1) treated with BFLA (50 nM) for 16 h. The cells were stained with TfR (green) and NS5A-specific (red) antibodies. Nuclei were stained with DAPI (blue). Laser intensity and digital gain were kept constant for untreated and BFLA-treated cells. Magnifications, x100. Scale bar represents 5 μm. **(C)** CLSM analysis of HCV-positive Huh7.5-Taxilin cells (Jc1) treated with BFLA (50 nM) for 16 h. The cells were stained with TfR (green) and NS5A-specific (red) antibodies. Nuclei were stained with DAPI (blue). Laser intensity and digital gain were kept constant for untreated and BFLA-treated cells. Magnifications, x100. Scale bar represents 5 μm. **(D)** Quantification of the fluorescence intensity of TfR in HCV-positive (Jc1) and HCV-negative (GND) Huh7.5 cells. Total fluorescence per cell was calculated using ImageJ software and the following formula: corrected total cell fluorescence (CTCF) = integrated density - (area of selected cell × mean fluorescence of background readings). In total, a minimum of 10 cells were measured (mean ± SEM). Two-tailed unpaired *t*-test, *p < 0.05. **(E)** Quantification of the fluorescence intensity of TfR in HCV-positive (Jc1) and HCV-negative (GND) Huh7.5-Taxilin cells. Total fluorescence per cell was calculated using ImageJ software and the following formula: corrected total cell fluorescence (CTCF) = integrated density - (area of selected cell × mean fluorescence of background readings). In total, a minimum of 10 cells were measured (mean ± SEM). Two-tailed unpaired *t*-test, ns = not significant.

To investigate the turnover of TfR in more detail, the subcellular localization of TfR was analyzed by confocal immunofluorescence microscopy. Huh7.5 and α-taxilin overexpressing Huh7.5 (Huh7.5-Taxilin) cells were electroporated with HCV RNA (Jc1) and treated with BFLA. When comparing HCV-positive to HCV-negative cells, a weaker TfR-specific signal was observed for the HCV-positive cells as compared to the control. Moreover, a more pronounced accumulation of TfR in dot-like structures can be observed for the HCV-positive cells, while in HCV-negative cells, TfR was almost evenly distributed (Figures 3A,B).

Bafilomycin A1 treatment leads in case of the HCV-negative cells to a moderate accumulation of the TfR that was much more pronounced in the HCV positive Huh7.5 cells (Figures 3A,B).

In case of the α-taxilin overexpressing cells (Huh7.5-Taxilin), a higher amount of the TfR was observed; however, the treatment with BFLA had no strong effect on the subcellular distribution/accumulation of the TfR. Moreover, in the α-taxilin overexpressing cells, there is no pronounced difference between HCV-positive and negative cells (Figure 3C).

To quantify the fluorescence signals, the CTCF values were determined for TfR in HCV-negative (GND) and HCV-positive (Jc1) Huh7.5 cells (Figure 3D) or in HCV-negative (GND) and HCV-positive (Jc1) Huh7.5-Taxilin cells; Figure 3E). The quantification shows that in HCV-positive cells, the TfR-specific signal is significant lower as compared to the GND cells. In case of the α-taxilin overexpressing Huh7.5 cells, there is no significant difference.

Taken together, these data further corroborate the observation that the decreased amount of α-taxilin in HCV-replicating cells leads to an impaired recycling of the TfR and increased lysosomal degradation, as reflected by the intracellular accumulation in case of BFLA treated cells. These data indicate that overexpression of α-taxilin in HCV-replicating cells supports the recycling of the TfR.

### TfR Colocalizes With LAMP-2 Positive Structures in HCV-Replicating Cells

**FIGURE 4:**
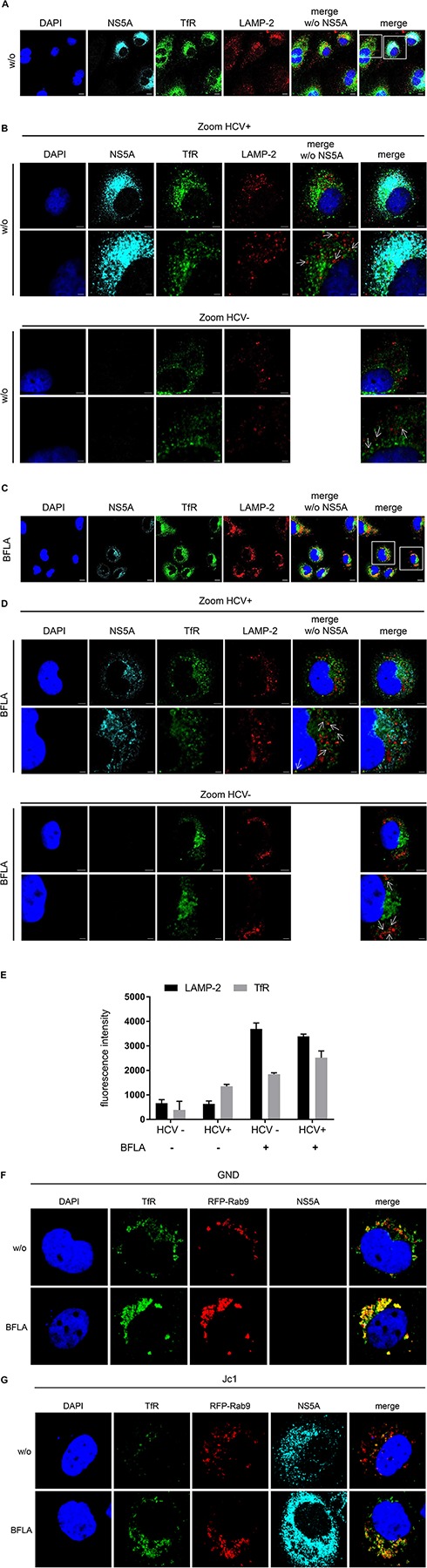
Colocalization of the TfR with LAMP-2 positive structures. **(A)** CLSM analysis of HCV-positive Huh7.5 cells (Jc1). The cells were stained using NS5A- (cyan), TfR- (green), and LAMP-2-specific (red) antibodies. Nuclei were stained with DAPI (blue). Laser intensity and digital gain were kept constant. Magnifications, x100. Scale bar represents 5 μm. **(B)** Higher magnification of the indicated areas (white squares) of **A** depicting HCV-positive (Jc1) and HCV-negative cells (GND). NS5A (cyan), TfR (green), and LAMP-2 (red). Nuclei were stained with DAPI (blue). Scale bar represents 5 μm for the upper lane and 2 μm for the lower lane, respectively. White arrows indicate the close association of TfR to the lysosomal marker LAMP-2. **(C)** CLSM analysis of HCV-positive Huh7.5 cells (Jc1) treated with BFLA (50 nM) for 16 h. The cells were stained using NS5A- (cyan), TfR- (green), and LAMP-2-specific (red) antibodies. Nuclei were stained with DAPI (blue). Laser intensity and digital gain were kept constant for untreated and BFLA-treated cells. Magnifications, x100. Scale bar represents 10 μm. **(D)** Higher magnification of the indicated areas (white squares) of **C** depicting HCV-positive (Jc1) and HCV-negative cells (GND) treated with BFLA (50 nM) for 16 h. NS5A (cyan), TfR (green), and LAMP-2 (red). Nuclei were stained with DAPI (blue). Magnifications, x100. Scale bar represents 5 μm for the upper lane and 2 μm for the lower lane. White arrows mark the close association of TfR to the lysosomal marker LAMP-2 after BFLA-treatment. **(E)** Quantification of the fluorescence intensity of TfR and LAMP-2 in lysosomal structures of untreated and BFLA-treated HCV-negative (GND) and HCV-positive cells (Jc1) (*n* = 2, mean ± SEM). **(F)** CLSM analysis of HCV-negative (GND) and **(G)** HCV-positive (Jc1) Huh7.5 cells. The cells were treated with BFLA (50 nM) for 16 h and stained using NS5A- (cyan) and TfR- (green)-specific antibodies; Rab9-RFP transfected cells are in red. Nuclei were stained with DAPI (blue). Laser intensity and digital gain were kept constant. Magnifications, x100.

To study the turnover of TfR in HCV-positive cells in more detail confocal double immunofluorescence microscopy was performed using TfR- and lysosome-associated membrane protein 2 (LAMP-2)-specific antibodies. Beside untreated cells, a treatment with BFLA was performed to trap the TfR in the lysosomal compartment (Figures 4A,C). Cells were zoomed in to visualize the close association of the TfR with LAMP-2-positive structures (Figures 4B,D). The fluorescence intensity of TfR in LAMP-2 positive lysosome-like spots was quantified and is shown in Figure 4E. The quantification of the immunofluorescence microscopy confirms a lower amount of TfR in HCV-positive cells; however, in lysosomal structures, there is a higher amount of TfR found as compared to these structures of HCV-negative cells (Figure 4E). To corroborate these data, Rab9 was used as a marker for the late endosomes (LEs). For this purpose, HCV-negative cells (GND) (Figure 4F) and HCV-positive cells (Jc1) (Figure 4G) were transfected with an expression vector encoding a Rab9-RFP fusion protein. The distribution of TfR was analyzed by a specific antibody (green fluorescence). The CLSM analysis revealed that a fraction of TfR colocalizes with Rab9. If Bafilomycin is present, an accumulation of TfR in the Rab9-positive structures can be observed. These data indicate that TfR has the capacity in Bafilomycin-treated and untreated cells to enter the LE/lysosomal compartment.

This reflects the impaired recycling of TfR in HCV-positive cells that targets the TfR to the autophagosomal-lysosomal compartment.

### Reduced Amount of the TfR on the Cell Surface in HCV-Replicating Cells

To investigate whether the impaired recycling of TfR in HCV-replicating cells (Jc1 cells) indeed leads to a reduced amount of TfR on the cell surface, surface biotinylation assays were performed. For this purpose, HCV-positive cells and as a control HCV-negative cells (GND cells) were labeled with the non-membrane permeable activated biotin derivate (EZ-link-sulfo-NHS-SS-biotin). This leads to the biotinylation of surface proteins while intracellular proteins are not labeled. The biotinylated proteins were precipitated using an avidin derivate that is coupled to agarose beads and analyzed by Western blot analysis.

**FIGURE 5:**
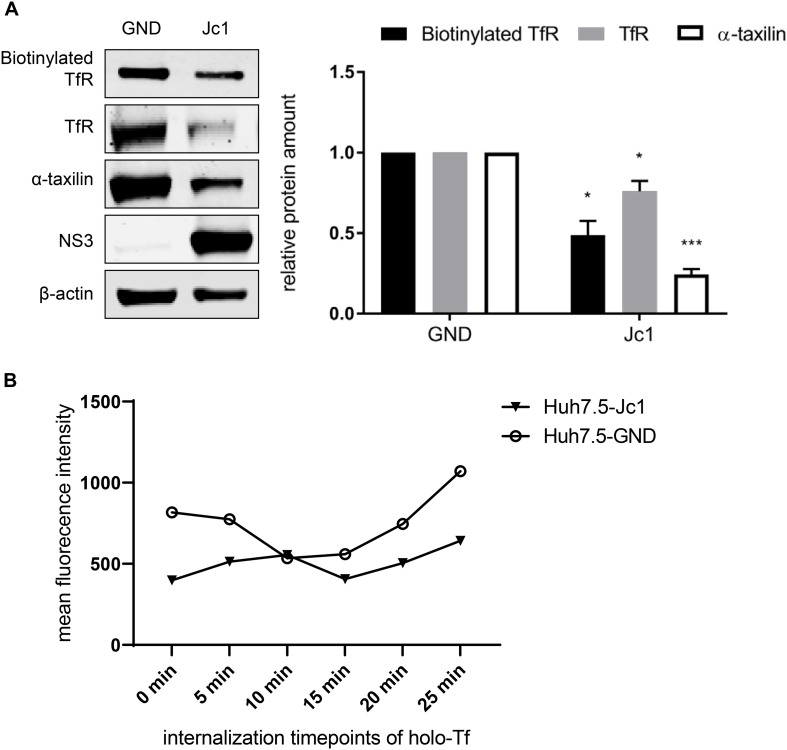
Reduced amount of TfR on the cell surface in HCV-replicating cells. **(A)** (Left) Western blot analysis of a biotinylation assay of cellular lysates derived from HCV-positive (Jc1) and HCV-negative Huh7.5 cells (GND). For biotinylation of surface proteins, the cells were treated with the non-membrane permeable activated biotin derivate EZ-link-sulfo-NHS-SS-biotin 72 hpe for 30 min, followed by precipitation with Pierce^TM^NeutrAvidin^TM^ agarose beads. Precipitated surface TfR was detected using a TfR-specific antibody and compared the total lysate of the corresponding cells. For detection of the total lysate, TfR-, α- taxilin-, and NS3-specific antibodies were used. ß-actin served as a loading control. (Right) Densitometric quantification of the biotinylated-TfR, the TfR, and α-taxilin in HCV-positive (Jc1) and HCV-negative cells (GND) from two independent experiments. Untreated HCV-negative cells (GND) served as a control and were set as 1 (*n* = 2 experiments; mean ± SEM). *p < 0.05, ***p < 0.001. **(B)** Mean fluorescence intensity of Huh7.5 electroporated with Jc1 or GND. After loading with holo-transferrin, cells were harvested an the amount of TfR on the cell surface of non-permeabilized cells was determined at different time points using a TfR-specific antibody that binds to the extracellular domain. The antibody was visualized by an Alexa488 conjugated secondary antibody and the analysis was performed by flow cytometry. One representative experiment is shown.

The Western blot analysis revealed that in case of the HCV-replicating cells significant less TfR is biotinylated. This indicates that less TfR is present on the surface of HCV-replicating cells (Figure 5A).

To monitor directly the effect of HCV on the recycling of TfR HCV-negative cells (GND) and HCV-positive cells (Jc1) were incubated with holo-transferrin for a defined time. After removal of the holo-transferrin cells were harvested over a time period of 25 min at defined time points. The amount of TfR at the different time points was determined by FACS analysis of non-permeabilized cells using a TfR-specific antibody that binds to the extracellular domain (Figure 5B). The recycling assay shows that in HCV-positive cells, less TfR is present on the surface.

Taken together these data indicate that the impaired recycling of TfR in HCV positive cells leads to a lower amount of TfR on the cell surface as compared to HCV-negative cells.

### Decreased Transferrin Binding and Iron Uptake in HCV-Replicating Cells

To further confirm that there is less TfR on the surface of HCV-replicating cells transferrin binding was analyzed. For this purpose, Huh7.5 cells were electroporated with the HCV RNA Jc1. AF488-labeled transferrin was added to the cells to study the binding/entry of transferrin in HCV-positive and negative cells. After removal of unbound AF488-labeled transferrin, confocal fluorescence microscopy was performed to quantify the amount of bound/internalized transferrin.

The AF488-transferrin-specific fluorescence was determined for HCV-positive cells and for HCV-negative cells at two time points, and fluorescence intensity was quantified using the Zen2009 software.

**FIGURE 6:**
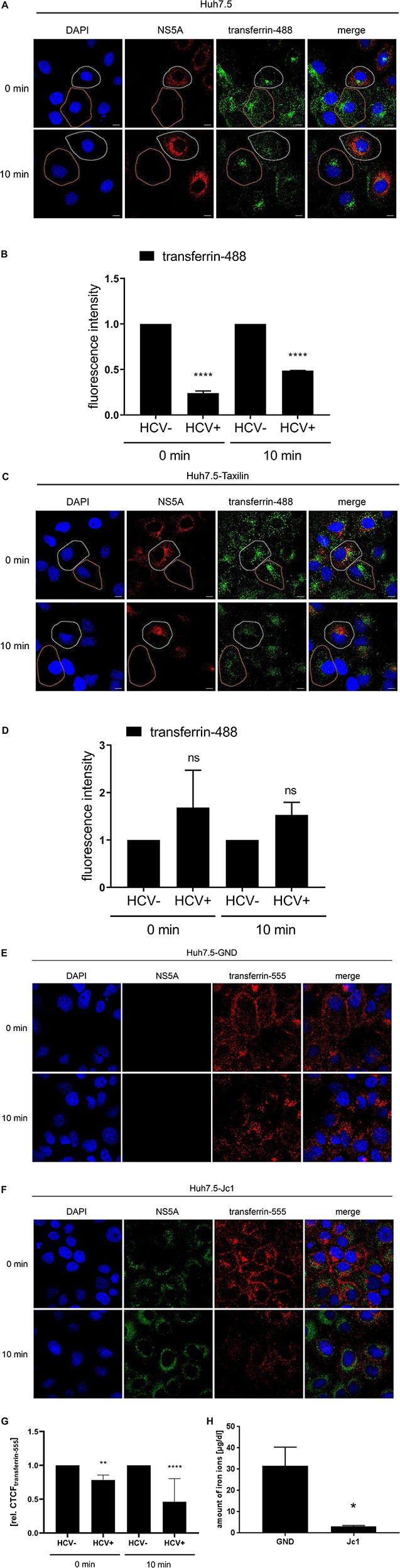
HCV impairs the recycling of the TfR is affected by HCV that can be rescued by α-taxilin overexpression. **(A)** CLSM analysis of HCV-positive Huh7.5 cells (Jc1), incubated with transferrin-488 (15 μg/mL) (green) for 1.5 h. After washing of unbound transferrin-488, the cells were immediately fixed or incubated for 10 min at 37°C to detect the internalized transferrin-488. HCV-positive cells were visualized using a NS5A-specific (red) antibody. Nuclei were stained with DAPI (blue). HCV-positive cell is indicated by a white spotted line and HCV-negative cell is indicated by an orange spotted line. Laser intensity and digital gain were kept constant. Magnifications, x100. The scale bars represent 10 μm. **(B)** Quantification of the fluorescence intensity of transferrin-488 in HCV-positive (Jc1) and HCV-negative (GND) Huh7.5 cells after 0 and 10 min incubation (*n* = 3, mean ± SEM). Two-tailed unpaired *t*-test, ****p < 0.0001. **(C)** CLSM analysis of HCV-positive (Jc1) Huh7.5-Taxilin cells incubated with transferrin-488 (15 μg/mL) (green) for 1.5 h. After washing of unbound transferrin-488, the cells were immediately fixed or incubated for 10 min at 37°C to detect the internalized transferrin-488. HCV-positive cells were visualized using a NS5A-specific (red) antibody. Nuclei were stained with DAPI (blue). HCV-positive cell is indicated by a white spotted line and HCV-negative cell is indicated by an orange spotted line. Laser intensity and digital gain were kept constant for untreated cells. Magnifications, x100. The scale bars represent 10 μm. **(D)** Quantification of the fluorescence intensity of transferrin-488 in HCV-positive (Jc1) and HCV-negative (GND) Huh7.5-Taxilin cells after 0 and 10 min incubation (*n* = 2, mean ± SEM). Two-tailed unpaired *t*-test, ns = not significant. CLSM analysis of **(E)** HCV-negative (GND) and **(F)** HCV-positive (Jc1) Huh7.5 cells, incubated with transferrin-555 (15 μg/mL) (red) for 1.5 h. After washing of unbound transferrin-555, the cells were immediately fixed or incubated for 10 min at 37°C to detect the internalized transferrin-555. HCV-positive cells were visualized using a NS5A-specific (green) antibody. Nuclei were stained with DAPI (blue). Laser intensity and digital gain were kept constant. Magnifications, x100. **(G)** Quantification of the fluorescence intensity of transferrin-555 in HCV-negative (GND) and HCV-positive (Jc1) Huh7.5 cells after 0 and 10 min incubation. Total fluorescence per cell was calculated using ImageJ software and the following formula: corrected total cell fluorescence (CTCF) = integrated density - (area of selected cell × mean fluorescence of background readings). In total, a minimum of 10 cells were measured (mean ± SEM). Two-tailed unpaired *t*-test, **p < 0.01, ****p < 0.0001. **(H)** Impaired uptake of iron ions in HCV-positive Huh7.5 cells (Jc1) compared to HCV-negative cells (GND) 72 hpe. The intracellular amount of Fe^2+^ was determined using a Cobas 8000 system (*n* = 3; mean ± SEM). Two-tailed unpaired *t*-test, *p < 0.05.

The quantification revealed for *t* = 0 min and for *t* = 10 min a significant lower signal for AF488-transferrin in the HCV-positive cells as compared to the HCV-negative cells (Figures 6A,B). In contrast to this, the quantification of the AF488-labeled-transferrin binding in Huh7-5-Taxilin cells led to a rescue of AF488-transferrin binding/entry in HCV-positive cells. This is reflected by a comparable fluorescence intensity at both time points comparing HCV-positive to HCV-negative cells (Figures 6C,D). The reduced fluorescence intensity in Huh7.5-Taxilin cells after 10 min indicates a higher turnover of the TfR and its ligand transferrin due to the α-taxilin overexpression. To exclude any unspecific effect that is due to the chromophore AF555-labeled transferrin was used in addition. The quantification revealed for *t* = 0 min and for *t* = 10 min a significant lower signal for AF555-transferrin in the HCV-positive cells as compared to the HCV-negative cells (Figures 6E–G).

This raises the question whether the significantly reduced transferrin binding/uptake is reflected by a change of the intracellular iron content in cell culture for HCV-positive cells as compared to negative cells. Determination of the intracellular iron level from HCV-replicating cells (Jc1) revealed a tenfold lower iron content of 3.1 μg/dL as compared to the HCV-negative control (GND) with 31.6 μg/dL iron ions (Figure 6H).

Taken together, these data indicate that the impaired TfR recycling in HCV-positive cells indeed leads to a reduced amount of TfR on the cell surface. This is reflected by a decreased transferrin binding/uptake leading to a lower iron content in HCV-replicating cells in cell culture.

### Double Infection of Hepatocytes Is Prevented by HCV

Hepatitis C virus-positive cells are poorly permissive for a superinfection by HCV. The data described above indicate that the impaired recycling of the TfR in HCV-positive cells has functional implications for binding of the ligand. As the TfR acts as coreceptor for HCV entry, the resulting question was whether the decreased amount of the TfR in HCV-replicating cells affects HCV (super)infection and thus is associated with an impaired double infection of HCV-replicating cells. To address this point, HCV-replicating cells were established by electroporation with the Jc1 E1R construct (Elgner et al., 2016b). HCV-replicating cells are characterized by the red fluorescence signal due to the formation of the mCherry tagged E1 protein. Two days after electroporation, the cells were infected with supernatant of Jc1 5AG-electroporated cells. In this case, HCV-replicating cells are characterized by a green fluorescence due to formation of a GFP-NS5A fusion protein.

**FIGURE 7:**
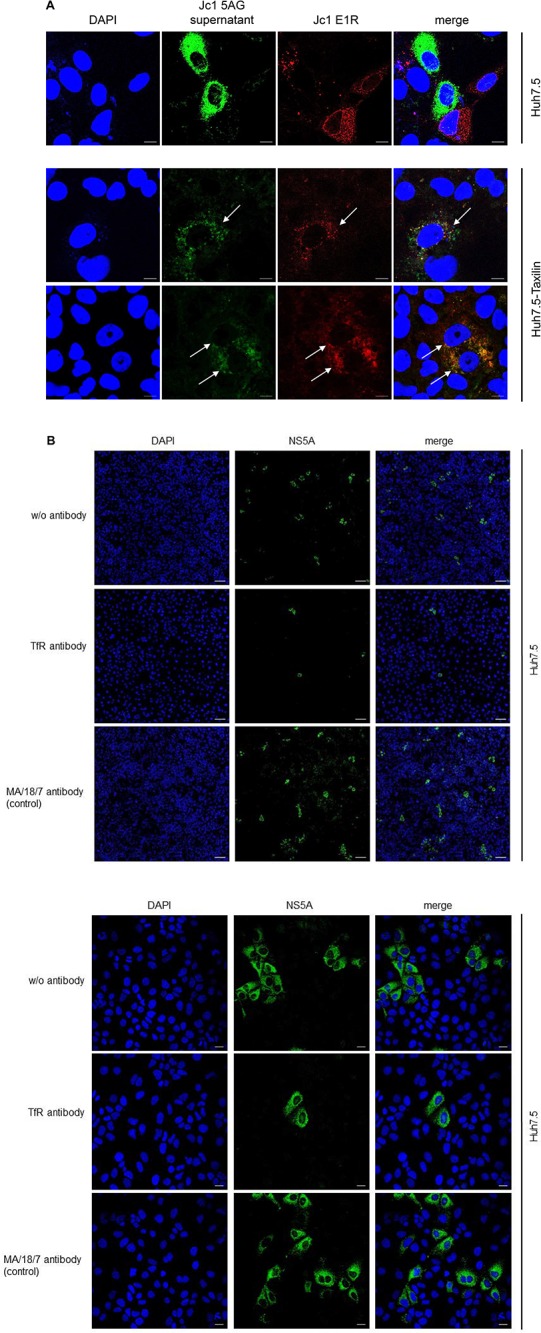
**(A)** Overexpression of α-taxilin overcomes block of superinfection (Top) CLSM analysis of HCV-positive Huh7.5 cell (Top) or stably α-taxilin overexpressing Huh7.5 cells (Huh7.5-Taxilin) (Bottom), electroporated with the Jc1 E1R construct, encoding for an E1-mCherry fusion protein (red). After 48 h, the cells were co-infected for 48 h with infectious Jc1 5AG supernatant, encoding for a NS5A-eGFP fusion protein (green). Double-positive cells are characterized by a yellow fluorescence signal and visualized by white arrows. Nuclei were stained with DAPI (blue). Laser intensity and digital gain were kept constant for untreated cells. Magnifications, x100. The scale bars represent 10 μm. **(B)** Neutralization of HCV infection with infectious supernatant by a TfR-specific antibody binding to the extracellular domain of TfR antibody. Incubation with the HBV-specific antibody MA18/7 served as control. Cells were stained using a NS5A-specific antibody (green). Nuclei were stained with DAPI (blue). Laser intensity and digital gain were kept constant. (Top) Magnifications, x10, the scale bars represent 100 μm. (Bottom) Magnifications, x40, the scale bars represent 20 μm.

Confocal fluorescence microscopy of Huh7.5 cells revealed either cells showing a red fluorescence or cells with green fluorescence, but no double positive cells characterized by a yellow signal were observed (Figure 7A, upper panel).

This is in accordance to previous data and indicates that an already existing HCV infection excludes a second infection by HCV (Schaller et al., 2007; Tscherne et al., 2007).

As described above in case of Huh7.5-Taxilin cells a rescue of the TfR recycling and a restauration of higher amounts of the TfR on the cell surface due to the α-taxilin overexpression was observed. Interestingly, superinfection of the HCV E1R replicating cells (red) by the Jc15AG virus (coding for GFP-NS5A) was possible as evidenced by cells simultaneously expressing the mCherry tagged E1 and the GFP-tagged NS5A (Figure 7A, lower panel).

To confirm the relevance of TfR for the HCV infection process in the Huh7.5 system, neutralization assays were performed. For this purpose, cells were preincubated with a TfR-specific antibody binding to the extracellular domain of TfR or with MA18/17 an HBV surface protein-specific antibody as control or were left untreated. Subsequent infection was performed in the presence of the respective antibodies. Detection of the infected cells by immunofluorescence microscopy using a NS5A-specific antiserum showed the strong inhibitory effect of the TfR-specific antibody on the HCV infection. This confirms the relevance of TfR for the HCV infection process (Figure 7B).

This indicates that the downmodulation of TfR on the surface of HCV-positive cells due to the decreased amount of α-taxilin is a causative factor for the block of superinfection. Rescue of the taxilin amount in HCV-replicating cells restores the level of TfR on the surface and overcomes the block of superinfection.

## Discussion

α-Taxilin was identified as a binding partner of syntaxin 1, 3, and 4 (Nogami et al., 2003, 2004). The binding is restricted to free syntaxin that is not part of the SNARE complex. Thus, α-taxilin exerts a negative regulatory function on the formation of SNARE complexes and thereby affects SNARE-dependent transport processes. Further analyses revealed that hepatitis viruses B and C affect the expression and amount of α-taxilin. While HBV replication leads to an increased expression and elevated amount of α-taxilin, the opposite is observed for HCV: expression and amount are decreased (Hoffmann et al., 2013; Elgner et al., 2016a). α-Taxilin affects the release of HCV by modulating the number of free syntaxin 4 molecules that can be used for formation of SNARE complexes. In accordance to this, an elevated amount of syntaxin 4 promotes the release of HCV, while silencing of the syntaxin 4 expression decreases the number of released viral particles (Ren et al., 2017). Moreover, overexpression of α-taxilin in HCV positive cells impairs HCV release while further silencing favors the release of infectious viral particles (Ren et al., 2017).

In a recent report, it was described that in HeLa cells α-taxilin interacts with SNX4 and thereby is involved in the recycling of the TfR (Sakane et al., 2014). A decrease in the amount of α-taxilin selectively impairs the recycling of TfR. Knockdown of α-taxilin leads to lysosomal degradation of TfR and impaired recycling of TfR. Further analyses revealed that SNX4 is involved in the recycling of TfR and interacts with α-taxilin (Sakane et al., 2014).

In our study, we could confirm for the hepatoma cell lines Huh7.5.1 and HepaRG that transient silencing or stable knockout of α-taxilin impairs the TfR recycling and thereby leads to a decreased amount of TfR. In light of the previous observation that in HCV-replicating cells the amount of α-taxilin is decreased (Elgner et al., 2016a), we investigated the impact on the recycling and amount of the TfR in HCV-positive cells. Indeed, the decreased amount of α-taxilin in HCV-positive cells correlates with a decreased level of the TfR that is not due to a decreased expression of the TfR, as the level of TfR-specific transcripts does not differ between HCV positive cells and the corresponding control. Inhibition of the lysosomal activity by BFLA increases the amount of the TfR in HCV-positive cells. This reflects that due to the impaired recycling TfR enters the lysosomal compartment. If the lysosomal activity is blocked by bafilomycin TfR-specific signals accumulate in the LE causing the enhanced TfR-specific signal under these conditions. Moreover, under these conditions, it can be observed that the TfR is trapped in LAMP-2-positive structures. As HCV induces autophagy, it can be assumed that this enhances the degradation of the TfR that is prevented from recycling by the lack of α-taxilin.

The HCV life cycle is strongly associated with autophagy (Medvedev et al., 2017). Inhibition of autophagy contributes to an inhibition of HCV replication. This could explain the reduced level of NS5A in HCV-positive Bafilomycin treated cells.

The impaired recycling of the TfR in HCV-positive cells that leads to a decreased amount of TfR can be rescued by α-taxilin overexpression, confirming the crosstalk between HCV, α-taxilin, and the TfR. The impaired recycling and subsequent lysosomal degradation of the TfR leads to a decreased total amount of the TfR and indeed to a decreased number of TfR molecules on the cell surface of HCV-positive cells. The functional relevance of this is reflected by the decreased binding capacity of AF488-labeled transferrin in HCV-positive cells. Again, overexpression of α-taxilin restores the transferrin binding capacity of HCV-positive cells.

The impaired recycling of TfR in HCV-positive cells due to a decreased level of α-taxilin is not a general phenomenon affecting the recycling/degradation of other receptors. The effect of α-taxilin depends on the interaction with SNX4 that is relevant for TfR internalization/recycling, but not a general adaptor that is involved in internalization/recycling processes. Silencing of α-taxilin expression does not lead to a decreased amount of EGFR. This is in accordance to the literature (Sakane et al., 2014) and our data.

The reduced amount of the TfR on the surface of HCV-positive cells that is associated with a decreased binding capacity of its ligand transferrin can be a causative factor that the amount of Fe^2+^ is significant lower as compared to the HCV-negative control cells. This fits to previous observations that describe a significant reduction of the iron content in HCV-positive cells as compared to the control cells (Fillebeen and Pantopoulos, 2013). In these previous studies, the authors describe an accumulation of iron regulatory protein 2 (IRP2) and report about a repression of TfR1 and divalent metal transporter in HCV-positive cells. This is corroborated by our data that confirm the decreased iron content in HCV-replicating cells and reveal a mechanism based on the decreased amount of α-taxilin leading to an impaired recycling and enhanced autophagosomal turnover. The decreased iron content promotes HCV replication as the viral RNA-dependent RNA polymerase NS5B is inhibited by elevated intracellular levels of Fe^2+^ (Fillebeen et al., 2005; Fillebeen and Pantopoulos, 2010).

The TfR was identified as a relevant HCV entry factor. It is well established that cells that are productively infected by HCV are refractory to a second infection by HCV known as superinfection exclusion. As there is clear evidence that superinfection exclusion is neither due to a reduction of CD81 and nor of SRB1, it is assumed that the block occurs at a post-entry step (Schaller et al., 2007). The TfR was identified as a relevant entry factor for HCV (Martin and Uprichard, 2013; Webster et al., 2013). Our data demonstrate that the reduced amount of α-taxilin in HCV-positive cells leads, via an impaired recycling, to a decreased number of TfR molecules that has functional implications on the transferrin binding and iron uptake. In light of the relevance of TfR for the HCV entry, the resulting question was whether the decreased TfR level on the cell surface could be a causative factor for the block of superinfection.

Superinfection experiments using either mCherry- (mCherryE1) or eGFP- (eGFPNS5A) tagged reporter viruses confirmed the superinfection exclusion in HCV-infected HuH7.5 cells. Either mCherry- or eGFP-positive cells but no double positive cells were detected. However, if the α-taxilin expression was restored the level of TfR on the cell surface of HCV-positive cells was rescued and the superinfection exclusion could be overcome, as evidenced by double infected cells replicating the mCherry- and eGFP-tagged reporter virus characterized by a yellow fluorescence signal. These data suggest that the decreased number of TfRs on the cell surface of HCV-positive cells due to the impaired recycling is a factor contributing to the superinfection exclusion. Restauration of the α-taxilin expression and thereby rescue of TfR presentation on the cell surface overcomes the superinfection block.

Taken together, our data establish a crosstalk between the TfR and α-taxilin that with respect to the HCV life-cycle was identified as a relevant factor affecting the release of HCV. Functional implications of this crosstalk are a reduced transferrin binding capacity and subsequent diminished iron uptake of HCV-positive cells. As overexpression of α-taxilin restores the number of TfRs on the cell surface and overcomes the block of superinfection, it is concluded that the α-taxilin-TfR crosstalk is a relevant factor for the superinfection exclusion.

This study contributes to deepen our knowledge about HCV life cycle and correlates a novel factor- α-taxilin so far being considered to affect HCV release- with impaired iron uptake and receptor down modulation.

It will be interesting to see in further studies, whether polymorphisms in the α-taxilin expression in patients correlate with superinfection.

## Data Availability Statement

All datasets generated for this study are included in the article/supplementary material.

## Author Contributions

VH, FE, JR, and DB performed the experiments. VH, FE, DB, and EH analyzed the data. VH, FE, JR, DB, and EH planned the experiments. VH, DB, and EH wrote the manuscript.

## Conflict of Interest

The authors declare that the research was conducted in the absence of any commercial or financial relationships that could be construed as a potential conflict of interest.

## Footnotes

1 http://n2t.net/addgene:62988
